# Supplementary material for: Antileishmanial Compounds Isolated from Psidium Guajava L. Using a Metabolomic Approach
Source: Molecules. 2019 Dec 11;24(24):4536. doi: 10.3390/molecules24244536 (PMC6943623; doi:10.3390/molecules24244536)
Supplement: Supplementary file 1 [file molecules-24-04536-s001.pdf]

Supplementary Materials from "Antileishmanial compounds isolated from Psidium guajava L. using a metabolomic approach"

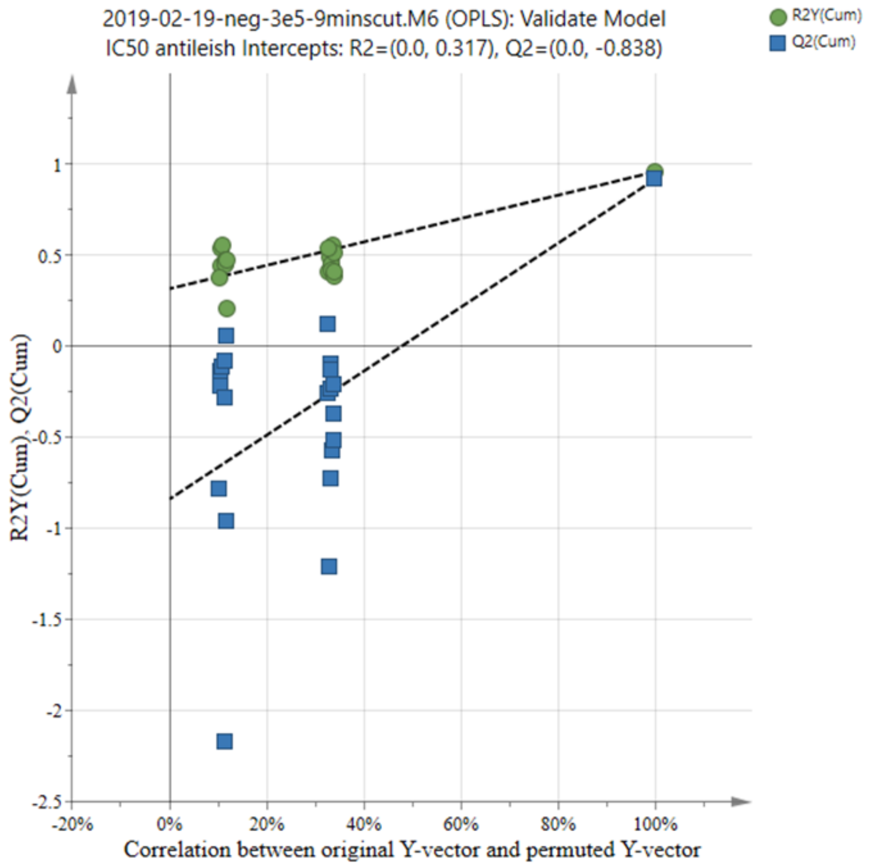

Figure S1. Permutation plot of the OPLS correlation model between antileishmanial activity and the liquid chromatography–mass spectrometry dataset.

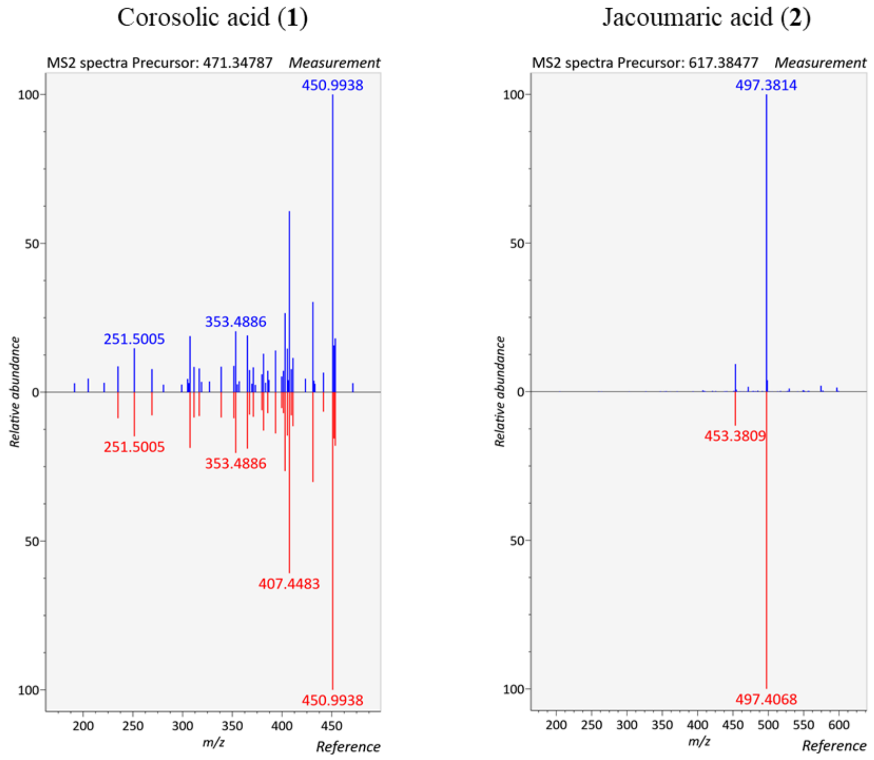

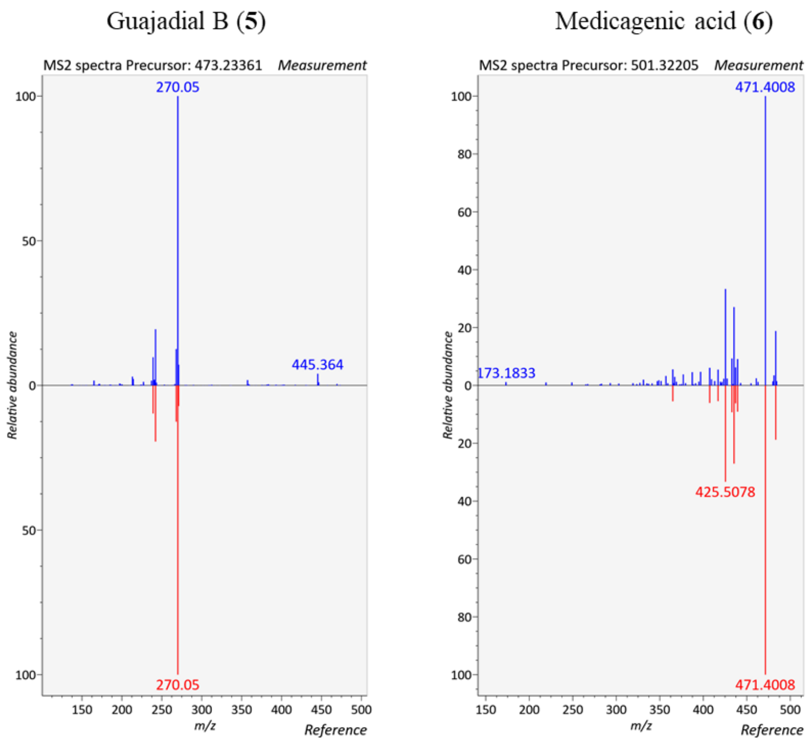

Figure S2. MS/MS fragmentation pattern of standard compound 1, 2, 5, 6 versus identified peaks in crude extract chromatograms.

Table S1. ^{1}H-NMR and ^{13}C-NMR data (DMSO-*d6*) of compound **1** (corosolic acid [39]).

No.	δ_H	δ_C	No.	δ_H	δ_C
1	1.80 m, 0.78 d (13.09)	47.55	18	2.12 d (11.31)	52.82
2	2.75 m	82.7	19	1.31	38.89
3	3.43 m	67.65	20	0.87	38.97
4		39.4	21	1.28, 1.44	30.62
5	0.78 d (13.1)	55.2	22	1.57	36.78
6	1.33, 1.47	18.48	23	0.93	29.26
7	1.28, 1.46	33.11	24	0.71 s	17.64
8		39.6	25	0.82 d (6.4)	17.48
9	1.5	47.44	26	0.75 s	17.41
10		38.04	27	1.05 s	23.75
11	1.87	23.39	28		178.77
12	5.16 dt (3.64, 15.38)	124.97	29	0.93	21.55
13		138.72	30	0.93	16.87
14		42.17	2-OH	4.27 dd (4.42, 6.90)	
15	0.99, 1.80	28.01	3-OH	4.38 d (4.13)	

16	1.53, 1.94	24.25	28-OH	11.95 S
17		47.29		

DMSO-*d6*: deuterated dimethyl sulfoxide.
